# Designing “Tiny Forests” as a lesson for transdisciplinary urban ecology learning

**DOI:** 10.1007/s11252-023-01371-7

**Published:** 2023-05-31

**Authors:** Monika Egerer, Michael Suda

**Affiliations:** 1 Department of Life Science Systems, School of Life Sciences, Technical University of Munich, Hans Carl-von-Carlowitz-Platz 2, 85354 Freising, Germany; 2 School of Management, Technical University of Munich, Hans Carl-von-Carlowitz-Platz 2, 85354 Freising, Germany

**Keywords:** Tiny Forests, Education for sustainable development, Urban forestry, Urban greening, Environmental education

## Abstract

The sustainability and livability of urban areas call for the next generation of scientists, practitioners and policy makers to understand the benefits, implementation and management of urban greenspaces. We harnessed the concept of "Tiny Forests©" – a restoration strategy for small wooded areas (~100-400 m^2^) – to create a transdisciplinary and experiential project for university forestry students that follows an ecology-with-cities framework. We worked with 16 students and a local municipality in the Munich, Germany metropolitan region to survey a community about its needs and desires and then used this information alongside urban environmental features and data collected by students (e.g., about soil conditions) to design a Tiny Forest. In this article, we describe the teaching concept, learning outcomes and activities, methodological approach, and instructor preparation and materials needed to adapt this project. Designing Tiny Forests provides benefits to students by having them approach authentic tasks in urban greening while experiencing the challenges and benefits of transdisciplinary communication and engagement with community members.

**Supplementary Information:**

The online version contains supplementary material available at 10.1007/s11252-023-01371-7.

## Introduction

Accessible greenspace is growing in importance in urban areas around the world. For example, many residents sought access to natural reserves or outdoor spaces within their neighborhood to engage in recreation and to de-stress during the COVID-19 pandemic (Kleinschroth and Kowarik 2020; Ugolini et al. 2020; Burnett et al. 2021), with the number of visits to forests nearby to cities increasing by 40% in some cases (Suda et al. 2021). Yet, forests where people can connect with nature may not be available within many urban landscapes. In response, cities are asking how they can design and implement greenspaces within a city’s built fabric that are accessible to all (Wolch et al. 2014; Bush 2017; Anguelovski et al. 2018; Tozer et al. 2020). Because space is often limited for public urban greenspace creation or transformation, new methods are needed to create or restore forests within urban areas that optimize the space for maximizing vegetation structure and biodiversity (Klaus and Kiehl 2021).

One such method is that of creating "Tiny Forests", initiated by Shubhendu Scharma, an industrial engineer turned eco-entrepreneur (^©^Afforesstt; see: https://www.afforestt.com/tinyforest) (Bleichrodt et al. 2017). The Tiny Forests concept is based on the 1970s restoration work of Japanese forester Akira Miyawaki (Lewis 2022). These forests are small – sometimes the size of a tennis court (100-150 m^2^) – but are meant to be "mighty" to allow for tall, dense forests and to bring natural elements closer to people (Afforesstt 2020). Tiny forests should have high species diversity and incorporate native species to address ecological restoration both from the perspective of forest function (e.g., habitat provision, temperature regulation) and landscape diversification. An important component of Tiny Forests is the involvement of local residents in the planning, implementation, and maintenance of the forests to ensure that their perspectives, wishes and needs for the design and function of nearby urban greenspaces are considered (Bleichrodt et al. 2017; Haringa 2020) *sensu* an ‘ecology with cities’ approach (Tanner et al. 2014; Bush 2017; Byrne 2022). In addition, universities play a vital role in training the next generation of scholars to understand and apply urban greening strategies like Tiny Forests to address social, ecological and technological urban challenges (Ulkhaq et al. 2018).

**Fig. 1:**
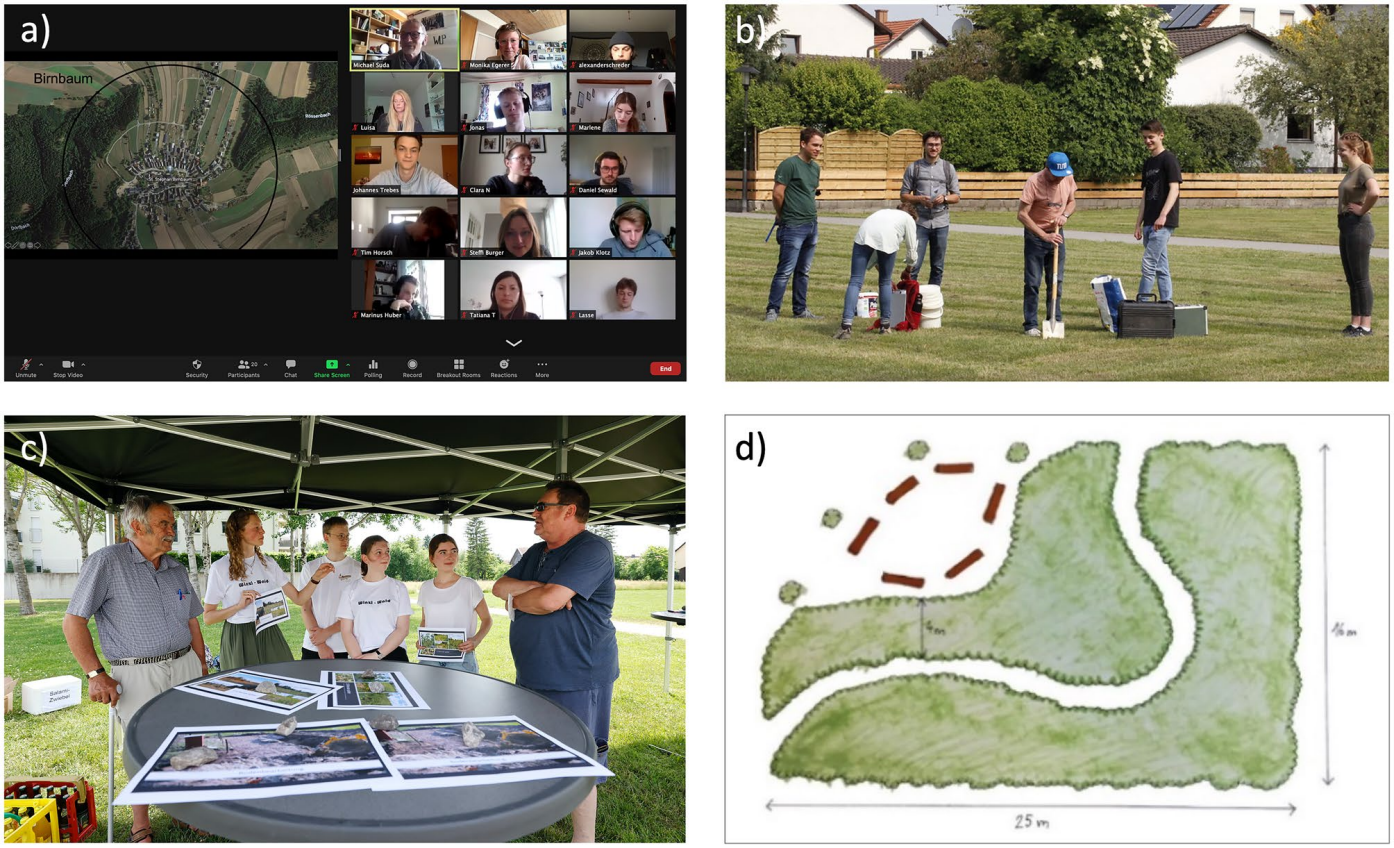
Snapshots of the teaching experience: **a**) first digital exercise in which students presented the greenspaces and nearby nature in their hometowns; **b**) sampling soils in the proposed sites in Hallbergmoos; **c**) community participation in “World Café” round table discussions documented by the local newspaper; d) one example of a proposed design of the overall structure of a Tiny Forest illustrated by the students. Pictures: Monika Egerer (**a**); Alexander Wegmann (**b**); and Marco Einfeldt (**c**)

We propose using Tiny Forests as a teaching tool for urban ecology and urban forestry education that helps advance many goals for Education for Sustainable Development (ESD) (UNESCO 2014) including integrating sustainability issues with vocational training; involving multi-stakeholder partnerships for high impact political engagement; and using interactive, learner-centered pedagogies. To those ends, we created a transdisciplinary Tiny Forests project for Bachelors Students in Forest Science and Management at the Technical University of Munich. Students gained first-hand ‘ecology-with-cities’ experience (Byrne 2022) through community engagement with the municipality of Hallbergmoos, Bavaria (a municipality of 11,000 inhabitants in the north of Munich, Germany) to explore the feasibility, benefits, and challenges of Tiny Forests planning and potential forest implementation in the region (Fig. 1). The goals of the project were to (1) develop a Tiny Forests plan together with community members and city authorities; (2) investigate residents’ perceptions, motivations and values in support of forest planning and implementation; and (3) build communication and project management skills of students through this real-life project with stakeholders (Khodeir 2018). Below, we provide an overview of this project to motivate and guide future transdisciplinary lessons on understanding social-ecological dimensions of small urban greenspaces, and translating that understanding into ecological design with community partners. This project can be adapted for other educational contexts and incorporate other learning activity examples and discussions.

### Learning outcomes

After completing this project, students should be able to:

- Identify the basics of project management using ‘backward design’ principles and how to plan and implement a project in a team using the example of designing Tiny Forests in urban environments.
- Implement ecological knowledge associated with the design and planting of Tiny Forests as related to soils, biodiversity, native plant species, etc.
- Justify forest design(s) including tree species selection and relationships with stakeholder perceptions and values.
- Effectively collaborate with stakeholders in the development and implementation of urban greening projects and communicate project outcomes to a non-scientific audience of community members and the general public.

### Course context

- Designed as an active learning project for 15-20 students, with second year undergraduates majoring in Forest Science and Resource Management and implemented across 10 weekly meetings; completed in partnership with stakeholders from the Mayor’s Office and a Sustainability Working Group in the neighboring municipality.
- Background information on urban forestry, urban planning, social science methods and ecosystem services is helpful for students to have prior to starting the project. This may come from their own prior academic experience or through materials provided by instructors as part of the course.
- The project was organized by a social scientist (forest and environmental politics) and a natural scientist (urban ecology), with support of a teaching assistant (Master’s student).
- Adaptable to other university contexts including introductory ecology, general environmental studies, and courses for non-science majors; larger class sizes are feasible but would likely require more organization and communication to coordinate multiple groups.

### Instructor preparation and materials

Instructors should solicit interest and agreement from a local municipality to be active stakeholders in the project. While the project could be done without such a partnership, it is recommended for helping students achieve the fullest learning gains because stakeholders can provide information about what outcomes they would like from a university-city partnership and other perspectives about concerns and context needed to inform planning such as the potential location(s) where Tiny Forests could be implemented.

To provide students with foundational context, background information should be gathered (e.g., some references listed in Supplementary Materials S1) and used to prepare an overview presentation with key concepts about the ecological and social benefits of urban greenspaces (forest, trees, parks, gardens, etc.). Instructors should specifically help students be able to (i) define greenspaces and the Tiny Forests concept and why they are important; (ii) describe the challenges to implement greenspaces in urban environments; (iii) lead a discussion of what might be done to improve urban planning and greenspace management; and (iv) introduce relevant information about the municipality and stakeholders. Some preparation will be required by instructors to gather materials and methods for site exploration and analyses. This could include city maps, satellite images and graphics of city landscapes and available sites, or any laboratory and field equipment to conduct soil sampling (see part 6 below and Box 2 for more context).

Invited guest speakers that e.g., have already implemented Tiny Forests, are experts in urban forestry, or are urban planners and landscape architects can help introduce theoretical and applied content such as the benefits and challenges of integrating urban ecosystem services (e.g., climate regulation, habitat provision, carbon sequestration), greenspace and relevant sociocultural issues and services (e.g., aesthetics, recreation, nature connection, social acceptance, cultural services) into urban planning and design. For example, we invited project leaders and initiators from around Germany to present examples of current Tiny Forests projects (e.g., ‘Urbane Waldgärten’, https://www.urbane-waldgaerten.de/; ‘Miya e.V.‘, https://www.miya-forest.de/). Outside voices can emphasize the relevance and timeliness of urban greening and can provide contemporary examples of Tiny Forests concepts; we see this as an important part of a transdisciplinary project where those who have non-academic experiences can help respond to student questions.

Box 1 We established four working groups of four students each based around the goals and associated tasks of the project (Fig. 3). Representatives of each of the working groups “networked” with another working group in interdisciplinary groups. The four groups included:

1. **Planning working group:** focused on the availability of greenspaces in the municipality, the land use history and different concepts for the Tiny Forest to present to residents. The students performed site assessments and analyzed soil samples to inform what trees could be planted.
2. **Survey working group**: developed a survey questionnaire that quantitatively assessed residents’ perceptions, motivations and values in relation to greenspace and biodiversity in the everyday lives of residents. Students led the design and organization of the community initiative, data collection and analysis.
3. **Community participation working group:** engaged residents in the planning process of the Tiny Forest to co-create a plan that represents community needs and values. To do so, students set-up a “World Café” round table event over a weekend to engage with residents in conversation using a qualitative approach. The World Café method was used to collect qualitative data on residents’ wants and needs for a Tiny Forest and greenspace.
4. **Communication working group:** worked with local newspapers, radio programs and university news to communicate the goals and events of the project. This group worked closely with each of the other groups to collect information on what each group was doing and, for example when events would occur in town.

Box 2 It is important to develop a “how-to-do” guide for implementing the Tiny Forest. In our example, we intentionally followed the five steps for planning Tiny Forests according to established and trademarked guidelines (https://www.ivn.nl/tinyforest/tiny-forest-worldwide) to provide students with a framework to work with and structure their project around; these steps can be discussed with students and provided to stakeholders during project communication. Note that these steps will likely not all be carried out in the project for a course (e.g. steps 4-5) but can nonetheless be discussed in lectures and meetings:

1. **Define natural vegetation community**. *What plants naturally grow on the site?* Surveys of the trees and shrubs in the sites should be conducted to know the current state and what species may need to be in the plans.
2. **Sample and understand the soil**. *What are soil conditions and how should the site be prepared for Tiny Forests?* Soil analyses may include texture, bulk density, organic matter, organic carbon, nutrients and pollutants, depending on resources available, including budgets to pay for analyses by analytical laboratories. Soil information provides the context for how the soil in the sites should be prepared to achieve loose nutrient-rich soil that has an increased water-holding capacity for optimizing plant growth. In the Tiny Forests protocol, when tilling the soil, the area is often dug down to about one meter and then mixed with straw. However, straw as a soil amendment is problematic in that it typically has a high carbon to nitrogen (C:N) ratio (80:1) and the recommendations are usually for materials that have a C:N ratio of between 20:1 and 30:1. Students can research a better soil amendment.
3. **Planting plan**. *What species should be planted, and how so?* Potential species compositions are provided and sketched out that distinguish main tree species, secondary tree species, and shrubs. Estimated costs of planting materials can be researched and provided within the plan, as this will be useful to stakeholders who would theoretically adopt and implement the plan with its associated costs.
4. **Planting day**. *How is planting carried out and who does the planting?* The area can be divided into a grid of one square meter each. One main tree species, one secondary tree species and one shrub are randomly planted per square. As many helpers are needed, projects often work in cooperation with schools.
5. **Maintenance**. *How should Tiny Forests be managed over time by stakeholders?* A recommendation is for the first 2-3 years and potentially beyond that the new forest will need nutrients and watering to reduce drought stress. Estimated time and resources should be calculated for the responsible stakeholder group.

## Learning activities (Fig. 2)

**Fig. 2:**
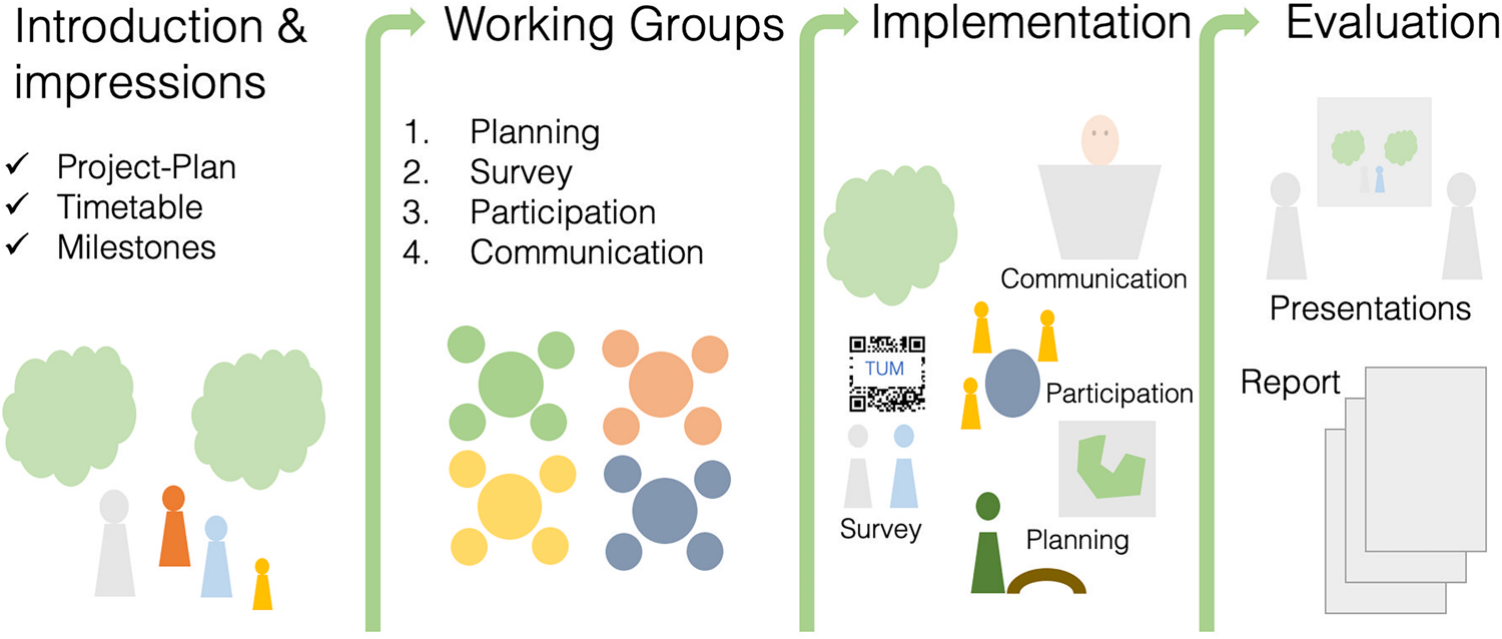
An overview of main steps of the project, including creation of the working groups (Fig. 3)

**Fig. 3:**
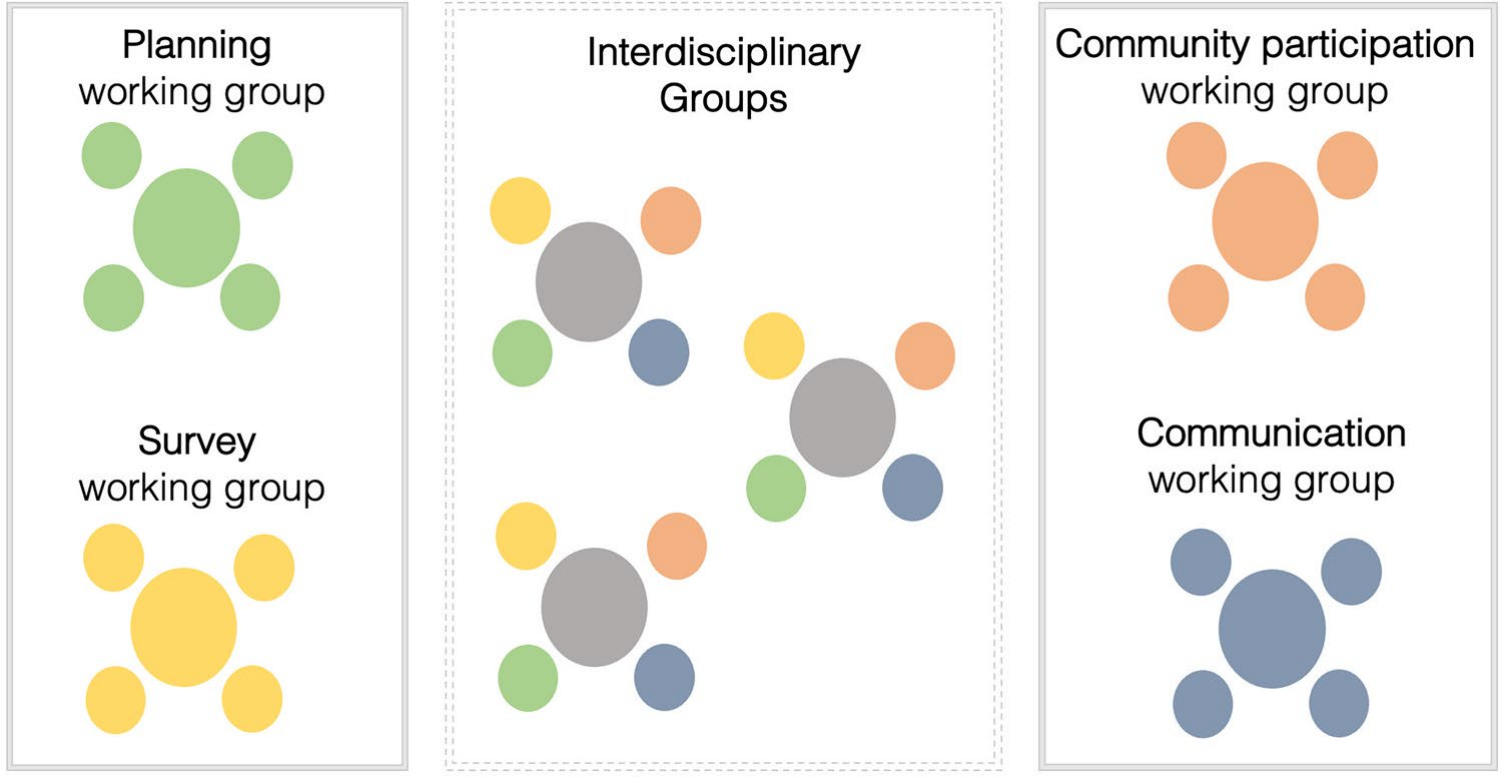
Schematic of the four working groups in the project (planning, survey, community participation, communication) of four students each, and how each member of the group also can work together in an interdisciplinary group with those of other groups. One person is a core organizer within each of the teams

- Part 1 (in person session; 60+ minutes): Introduce urban forestry and urban greening concepts including Tiny Forests in a short lecture and lead a discussion on the importance of greenspaces for ecosystem services and human wellbeing. Identify and explain research and applied gaps in urban forestry that are relevant to the creation and maintenance of a Tiny Forest (e.g., tree species selection, cooling benefits, social values around forests), which will ultimately guide project tasks and planning processes. For example, a lecture can discuss future urban climate conditions and the open question of which tree species may survive in hotter cities (Farrell et al. 2022; Sousa-Silva et al. 2023), or how urban residents associate different values – both positive and negative – with city trees and their traits (Ordóñez et al. 2016; Ordóñez Barona et al. 2022).
- Part 2 (in person session; 120+ minutes): To support students’assessment of the heterogeneous ‘green’ spaces – and lack thereof – in urban landscapes, ask students to identify urban greenspaces around their hometowns, specifically public lands and nearby nature, and hypothesize specific benefits of each, e.g., for biodiversity conservation, recreation, and aesthetics. We have found this to be a valuable exercise for increasing the relevance of the project by anchoring it in students’ personal histories and experiences and (re)connecting them with potential spaces that played important roles in their pasts.
- Part 3 (in person session, field trip, and/or individual work; 120+ minutes): Introduce students to the location(s) of the Tiny Forests. This could either consist of simply showing it to them via slides and maps during a session (which the instructor would prepare in advance), visiting the location together as a whole group via shared transportation, or asking students to visit the location and take photos of the area for which the Tiny Forest will be designed on their own. The students should produce an initial assessment and opinions of the area’s greenspace availability and access based on questions such as: is there abundant greenspace for people to visit and recreate in? What is the diversity and structural characteristics of trees and shrubs? Who is using the spaces and what activities are they doing?
- Part 4 (in person session; 90+ minutes): Have students present their photos and assessment notes to the class with associated discussion and synthesis as desired. The instructor should introduce an outline for project management for planning of the Tiny Forest through working “backward” – starting with the project end goal(s) and then preparing a project timeline that sets tasks, milestones and goals (see Box 2 for an outline of key steps) (Pope-Ruark 2012; Reynolds and Kearns 2016; Magano et al. 2021).
- Part 5 (in person session; 60+ minutes): Create working groups for different aspects of the project based on students’ interests, personalities and/or expertise (e.g., urban forestry, urban planning, social science, science communication). In our case, we created four working groups that we thought represent key components of the project and process, but these could be easily adapted by instructors. The four groups of four students focused on: (1) planning and design of Tiny Forests; (2) social science investigation of community wants and needs; (3) community participation for greenspace planning); and (4) public relations and communication (Fig. 3; see Box 1 for full descriptions). Individual group work should be emphasized, where students focus on tasks within their respective groups; however, the success of individual group work should also require students to communicate with the other groups. For example, the ‘planning’ group would need to know what kinds of trees (native, edible, flowering) residents would prefer in a Tiny Forest, which both the ‘community participation’ and ‘survey’ groups would determine. To facilitate this between-group exchange, a communication network within and among groups can be established. In our case, we assigned one group member to be a contact person to one of the other three groups, with the fourth as that group’s core person or leader, who facilitated the communication and coordination of the group. In addition, each of the instructors served as the primary mentor for two groups to facilitate advising tasks and meeting project goals.
- Part 6 (outside of in person sessions; 120+ minutes): To guide forest planning and installation decisions, students need to know current conditions of the site and prepare information for site preparation that may be necessary. For example, soil conditions should be investigated to know what trees could be planted, or whether soil fertility would need to be improved; the underground gray infrastructure could be researched (water pipes, internet cables, etc.) to determine if anything might impede or influence tree planting location or tree selection. In addition, knowing what species are already present at the site is important to integrate into forest design. For this, students should (1) collect and analyze soil samples (Box 2) to characterize textures to characterize conditions that inform tree selection and (2) inventory tree species richness and sizes (diameter at breast height; DBH) within the site(s). For the inventory, students can create simple sketches of the site to document tree and locations.
- Part 7 (in person session, group work, ~3 additional group meetings and events; 320+ minutes): Students should develop a survey questionnaire with support from instructors and distribute it in the municipality, e.g., using posters with QR-Codes to the online survey that are hung in public spaces and announcements in local newspapers (Fig. 1c; our students’ survey questionnaire and results are provided in the Supplementary Information S2 and S3). Students might also be guided to host a participatory ‘World Café’ event to gather information about local residents’ perceptions, needs and wants by engaging them through structured conversation in which groups discuss various topics at different tables with a group facilitator (Fig. 1d; collective knowledge sharing, Löhr et al. 2020). In our case, the World Café was organized by the community participation working group; they printed flyers and advertised the event in the local newspapers, rented out tables and tents to set up in open greenspaces, and provided refreshments. They prepared questions about greenspace use, photos of potential trees and shrubs that could be planted, and photos of Tiny Forests to help collect qualitative data via conversations with participants (about 15 people).
- Part 8 (optional in person sessions; 45 minutes each): Invite guest speakers to present topics related to urban forestry, urban greening and urban ecology that may be relevant to Tiny Forests, as well as to discuss challenges and opportunities. As possible, students can share their preliminary results and thoughts about the project with the guest speakers.
- Part 9 (group work, in person session, optional presentation to stakeholders; 120+ minutes): Each working group prepares a final written report and accompanying oral presentation of their results and conclusions. The final written reports should include a section reflecting on the group work within each group and across the groups. The length and format of both the written report and oral presentation can be tailored by instructors. In addition, both could be formatted required to be communicated to community partners and stakeholders as a form of authentic assessment (Villarroel et al. 2017).

## Extensions and additional connections

Additional activities or topics could be added or expanded upon, depending on the direction and emphasis of the course and how much time is available. Some suggestions include:

- Prepare pamphlets and/or presentations for stakeholders (e.g., municipality officials, community partners and/or local residents) to summarize the project and describe general steps to creating a Tiny Forest that is easy to understand for lay audiences (Box 2; Supplementary Information S4). Again, this can be a useful form of authentic assessment, in which tasks and performance standards are contextualized within (and mimic those) in the real world of work (Villarroel et al. 2017).
- Study the change of greenspace over time and analyzing travel times between existing greenspaces as ways to justify the need for a Tiny Forest in an area (Meerow and Newell 2017).
- Investigate the process of obtaining permission to plant Tiny Forests. Who needs to be involved, and what issues arise? This can be an opportunity to discuss policies and regulations about greenspaces and urban greening projects (e.g., zoning regulations, private versus public land use).
- Explore how selecting different tree species creates trade-offs between ecosystem services and disservices (Roman et al. 2021). For example, some tree species offer aesthetic and provisioning benefits (e.g., apple), while others cause allergies (e.g., hazel) (Lyytimäki et al. 2008; Lyytimaki and Faehnle 2009; Lyytimäki 2017).
- Evaluate the climate resilience of native versus non-native tree and shrub species. What species may be winners or losers of a warming climate in urban contexts (Ossola et al. 2020; Marchin et al. 2022)?
- Discuss concepts of urban nature. What is nature? How do we define what is and what is not nature? What form(s) of urban nature are associated with Tiny Forests (Kowarik 2011)?
- Reflect on environmental justice and green gentrification to address questions such as, who has access to (small) urban greenspaces and what are the equity and ethical challenges of urban greening (Wolch et al. 2014)?

## Reflections and conclusions

Project-based, authentic, and experiential activities are needed in higher education for learners to gain a comprehensive understanding of transdisciplinary approaches in urban greening initiatives due to the diversity of stakeholders (residents, city government officials) involved in projects (Cole 2010; Eilam and Trop 2010; Brewer et al. 2015). Environmental and social science concepts and methods can help learners approach contemporary global challenges with diverse, collaborative and inclusive perspectives (Payne and Jesiek 2018). The forest restoration strategy ‘Tiny Forests’, which is increasingly adopted by urban forestry and urban greening projects around the world (Thornton 2020; Straehler-Pohl 2021), provides a transdisciplinary, unique and graspable concept to teach students about the benefits of urban forests and greenspaces – even if small in area – and to build competence and confidence in project organization and science communication skills. In addition, such lessons allow instructors from different disciplines (in our case, urban ecology and forest policy) to pursue interdisciplinary collaboration and gain mutual appreciation of different natural and social science approaches in teaching. The utilization of this concept for a teaching activity could be adapted to other contexts including service-learning courses or single lectures, or short 1-day exercises that have students work with satellite imagery and tree species lists for urban areas without stakeholder engagement.

The Tiny Forests project positioned students directly in a real-world situation to engage with community members, through which they gained competency in urban forestry practices, and project management that they will need in future careers. Students had to set a goal and schedule, define tasks and milestones, and coordinate the activities of individual subgroups. Several students rose as leaders within their groups and this project has inspired them to continue in urban forestry, science communication, and public engagement in conservation. Successful communication and publication in news media outlets including local newspapers (Süddeutsche Zeitung, https://www.sueddeutsche.de/muenchen/freising/ein-winzl-wald-fuer-hallbergmoos-die-buerger-wuenschen-sich-mehr-gruen-1.5406672; Mooskurier, https://www.mooskurier.de/21/06/2021/ein-mini-wald-hinterm-rathaus/) provided external gratification to the students that the project was relevant to society and global issues. Furthermore, although our project was limited to planning and did not result in creation of an actual Tiny Forest, it nevertheless involved residents in the process by giving them a platform to communicate their needs and wants. Residents that engaged in the World Café were very positive and interested in the project, with hopes that one day the project would be implemented. For students, they reflected that the communication with local residents provided an authentic experience that gave them new perspectives beyond the theoretical aspects of a university education.

We identified a few challenges to implementing such a project in a university context and in an existing community. First, in our case, obtaining permissions from the city for where the students could actually conduct the study was difficult, though necessary for providing a place where soil conditions and other environmental characteristics could be evaluated to inform the planning process. Discussing realistic locations or wishes for Tiny Forests with residents was also challenging. Furthermore, while our community liaison was relatively communicative with us instructors, they did not effectively communicate with the city Mayor’s Office about all steps of the project, which was important in the case of advertising and conducting the World Café in town. This provided a learning opportunity, as the students gained insights on how administration and political bodies function. For project communication and translation, we recommend that communities with a relatively engaged and well-connected liaison can enrich the learning experience. Additionally, because Tiny Forests are a new strategy being discussed in Germany (Straehler-Pohl 2021), we had few real-world examples that have already been implemented for students for to critically analyze. This meant that most of our analysis of Tiny Forests benefits was largely theoretical, and discussions of Tiny Forests implementation were limited to few and very new case studies where long-term results (often the proposed justification for forest implementation) are missing. For students, this meant that they had little evidence with which to learn by example and thereby justify their Tiny Forests concepts. Another key consideration is that students had to be highly self-motivated and organized to maintain momentum and achieve project milestones within a short time frame of three months. Unfortunately, communication among working groups was lacking, which hindered linking the project components. Collectively these challenges can be included in classroom discussions so that students can reflect on them as part of the learning process.

In conclusion, urban areas are a contemporary frontier for education for sustainable development. Urban greening and urban forestry concepts are increasingly more dynamic, combining new approaches in nature-based solutions with old principles in restoration and forest ecology, as exemplified by Tiny Forests. Yet, to realize a more sustainable future, instructors must also provide real-world learning projects that help train the next generation of researchers, practitioners and policy makers in managing complex problems. Tiny Forests are a teaching theme that immerse students in science and social engagement to see the urban forest as more than just an individual tree, but as a collective endeavor to achieve sustainable development goals in growing, yet hopefully greener built environments.

## Supplementary Information

Below is the link to the electronic supplementary material.

Supplementary file1 (DOCX 10913 KB)

## Data Availability

All data available upon request.
