# Supplementary material for: Designing “Tiny Forests” as a lesson for transdisciplinary urban ecology learning
Source: Urban Ecosyst. 2023 May 31:1–9. Online ahead of print. doi: 10.1007/s11252-023-01371-7 (PMC10230492; doi:10.1007/s11252-023-01371-7)
Supplement: Supplementary file 1 — Supplementary file1 (DOCX 10913 KB) [file 11252_2023_1371_MOESM1_ESM.docx]

**Supplementary Information**

**Supplementary Information** **1**. Resources around Tiny Forests useful to teaching preparation and for students self-learning.

**Books:**

Barbosa, P. (2020) *Urban Ecology: Its Nature and Challenges*. CAB International, Boston, MA.

Ferrini, F., van den Bosch, C.C.K. & Fini, A. (2017) *Routledge Handbook of Urban Forestry*, 1st Edition. Taylor and Francis Inc.

Lewis, H. (2022). *Mini-Forest Revolution: Using the Miyawaki Method to Rapidly Rewild the World*. Chelsea Green Publishing.

﻿Marzluff JM, Shulenberger E, Endlicher W, et al (2008) *Urban Ecology*. Springer, New York.

**Reports:**

Castro, J., Ostoić, S.K., Cariñanos, P., Fini, A. & Sitzia, T. (2018) *‘Edible’ Urban Forests As Part of Inclusive, Sustainable Cities*. ﻿Unasylva 250: 69.

﻿Haringa J (2020) How just are civil society nature initiatives? Research on (spatial) injustice of Tiny Forests in the Netherlands. Radboud Universiteit

Jay, M., Selter, A., Wurster, M,. Schraml, U., (2016): Urbaner Wald, urbane Lebensqualität. Die vielfältigen Ökosystemleistungen urbaner Wälder sichtbar machen – Ein Handlungsleitfaden. Arbeitsbericht 01/2016, Professur für Forst- und Umweltpolitik, Albert-Ludwigs-Universität Freiburg. <https://www.forstpolitik-umweltpolitik.uni-freiburg.de/publikationen-1/arbeitsberichte/01_2016>

Ottburg et al. (2018). *Tiny Forest Zaanstad: citizen science and determining biodiversity in Tiny Forest Zaanstad*. Wageningen. <https://doi.org/10.18174/446911>

﻿Funding trees for health: an analysis of finance and policy actions to enable tree planting for public health. R. McDonald, L. Aljabar, C. Aubuchon, H.G. Birnbaum, C. Chandler, B. Toomey & J. Daley, et al. 2017. Arlington, USA, The Nature Conservancy.

**Journal articles:** a selection of articles that may be useful for teaching and providing to students, divided into general categories; note that some articles may certainly fit into other categories.

***Urban forests:***

Avolio, M.L., Pataki, D.E., Pincetl, S., Gillespie, T.W., Jenerette, G.D. & McCarthy, H.R. (2015) Understanding preferences for tree attributes: the relative effects of socio-economic and local environmental factors. *Urban Ecosystems*, **18**, 73–86.

Alvey, A.A. (2006) Promoting and preserving biodiversity in the urban forest. *Urban Forestry & Urban Greening*, **5**, 195–201.

Frank, S.D., Backe, K.M., McDaniel, C., Green, M., Widney, S. & Dunn, R.R. (2019) Exotic urban trees conserve similar natural enemy communities to native congeners but have fewer pests. *PeerJ*, **7**, e6531.

Jasmani, Z., Ravn, H.P. & van den Bosch, C.C.K. (2017) The influence of small urban parks characteristics on bird diversity: A case study of Petaling Jaya, Malaysia. *Urban Ecosystems*, **20**, 227–243.

Jenerette, G.D., Clarke, L.W., Avolio, M.L., Pataki, D.E., Gillespie, T.W., Pincetl, S., Nowak, D.J., Hutyra, L.R., McHale, M., McFadden, J.P. & Alonzo, M. (2016) Climate tolerances and trait choices shape continental patterns of urban tree biodiversity. *Global Ecology and Biogeography*, **25**, 1367–1376.

Ordóñez Barona, C., Wolf, K., Kowalski, J.M., Kendal, D., Byrne, J.A. & Conway, T.M. (2022) Diversity in public perceptions of urban forests and urban trees: A critical review. *Landscape and Urban Planning*, **226**.

Ossola, A., Hoeppner, M.J., Burley, H.M., Gallagher, R. V., Beaumont, L.J. & Leishman, M.R. (2020) The Global Urban Tree Inventory: A database of the diverse tree flora that inhabits the world’s cities. *Global Ecology and Biogeography*, 1–8.

Rahman, M.A., Hartmann, C., Moser-Reischl, A., von Strachwitz, M.F., Paeth, H., Pretzsch, H., Pauleit, S. & Rötzer, T. (2020) Tree cooling effects and human thermal comfort under contrasting species and sites. *Agricultural and Forest Meteorology*, **287**, 107947.

Romanova, O. & Lovell, S. (2021) Food safety considerations of urban agroforestry systems grown in contaminated environments. *Urban Agriculture & Regional Food Systems*, **6**, e20008.

Schwaab, J., Meier, R., Mussetti, G., Seneviratne, S., Bürgi, C. & Davin, E.L. (2021) The role of urban trees in reducing land surface temperatures in European cities. *Nature Communications 2021 12:1*, **12**, 1–11.

Vogt, J. (2020) Urban Forests as Social-Ecological Systems. *Encyclopedia of the World’s Biomes*, pp. 58–70. Elsevier.

***Urban planning and policy:***

Fernandes, C.O., da Silva, I.M., Teixeira, C.P. & Costa, L. (2019) Between tree lovers and tree haters. Drivers of public perception regarding street trees and its implications on the urban green infrastructure planning. *Urban Forestry & Urban Greening*, **37**, 97–108.

Green, O.O., Garmestani, A.S., Albro, S., Ban, N.C., Berland, A., Burkman, C.E., Gardiner, M.M., Gunderson, L., Hopton, M.E., Schoon, M.L. & Shuster, W.D. (2015) Adaptive governance to promote ecosystem services in urban green spaces. *Urban Ecosystems*.

Mcdonald, R.I., Aronson, M.F.J., Beatley, T., Beller, E., Bazo, M., Grossinger, R., Jessup, K., Andressa, M., Puppim De Oliveira, J.A., Panlasigui, S., Burg, J., Pevzner, N., Shanahan, D., Stoneburner, L., Rudd, A. & Spotswood, E. (2022) Denser and greener cities: Green interventions to achieve both urban density and nature. *People and Nature*, **00**, 1–19.

Pettorelli, N., Barlow, J., Stephens, P.A., Durant, S.M., Connor, B., Schulte to Bühne, H., Sandom, C.J., Wentworth, J. & du Toit, J.T. (2018) Making rewilding fit for policy. *Journal of Applied Ecology*, **55**, 1114–1125.

Sousa-Silva, R., Duflos, M., Barona, C. O., & Paquette, A. (2023). Keys to better planning and integrating urban tree planting initiatives. *Landscape and Urban Planning*, 231, 104649.

﻿Tanner CJ, Adler FR, Grimm NB, et al (2014) Urban ecology: advancing science and society. *Front Ecol Environ* 12:574–581. doi: 10.1890/140019

***Ecosystem services and disservices:***

Czaja, M., Kołton, A. & Muras, P. (2020) The Complex Issue of Urban Trees—Stress Factor Accumulation and Ecological Service Possibilities. *Forests 2020, Vol. 11, Page 932*, **11**, 932.

Lin, B.B., Meyers, J., Beaty, R.M. & Barnett, G.B. (2016) Urban green infrastructure impacts on climate regulation services in Sydney, Australia. *Sustainability*, **8**, 1–13.

Roman, L.A., Conway, T.M., Eisenman, T.S., Koeser, A.K., Ordóñez Barona, C., Locke, D.H., Jenerette, G.D., Östberg, J. & Vogt, J. (2020) Beyond ‘trees are good’: Disservices, management costs, and tradeoffs in urban forestry. *Ambio*, 1–16.

Rötzer, T., Moser-Reischl, A., Rahman, M.A., Hartmann, C., Paeth, H., Pauleit, S. & Pretzsch, H. (2021) Urban tree growth and ecosystem services under extreme drought. *Agricultural and Forest Meteorology*, **308**–**309**, 108532.

Shah, A.M., Liu, G., Huo, Z., Yang, Q., Zhang, W., Meng, F., Yao, L. & Ulgiati, S. (2022) Assessing environmental services and disservices of urban street trees. an application of the emergy accounting. *Resources, Conservation and Recycling*, **186**.

***Environmental justice:***

Carmichael, C.E. & McDonough, M.H. (2018) The trouble with trees? Social and political dynamics of street tree-planting efforts in Detroit, Michigan, USA. *Urban Forestry & Urban Greening*, **31**, 221–229.

Kato-Huerta, J. & Geneletti, D. (2022) Environmental justice implications of nature-based solutions in urban areas: A systematic review of approaches, indicators, and outcomes. *Environmental Science & Policy*, **138**, 122–133.

Lin, B.B., Meyers, J. & Barnett, G. (2015) Understanding the potential loss and inequities of green space distribution with urban densification. *Urban Forestry & Urban Greening*, **14**, 952–958.

Poe, M.R., McLain, R.J., Emery, M. & Hurley, P.T. (2013) Urban Forest Justice and the Rights to Wild Foods, Medicines, and Materials in the City. *Human Ecology*, **41**, 409–422.

Schwarz, K., Fragkias, M., Boone, C.G., Zhou, W., Mchale, M., Grove, J.M., Neil-dunne, J.O., Mcfadden, J.P., Buckley, G.L., Childers, D., Ogden, L., Pincetl, S. & Pataki, D. (2015) Trees grow on money: urban tree canopy cover and environmental justice. *PLoS ONE*, **10**, 1–17.

Wolch, J.R., Byrne, J. & Newell, J.P. (2014) Urban green space, public health, and environmental justice: the challenge of making cities ‘just green enough’. *Landscape and Urban Planning*, **125**, 234–244.

***Participatory approaches, social science, etc.***

﻿Colding J, Barthel S (2013) The potential of “Urban Green Commons” in the resilience building of cities. Ecol Econ 86:156–166. doi: 10.1016/j.ecolecon.2012.10.016

﻿﻿

Hunter AJ, Luck GW (2015) Defining and measuring the social-ecological quality of urban greenspace: a semi-systematic review. Urban Ecosyst. doi: 10.1007/s11252-015-0456-6

Fischer LK, Kowarik I (2020) Dog Walkers’ Views of Urban Biodiversity across Five European Cities. Sustainability 12:3507. doi: 10.3390/su12093507

McNamara, K.A., Kostelny, M., Kim, G., Keating, D.M., Estiandan, J. & Armbruster, J. (2022) A novel resident outreach program improves street tree planting outcomes in Los Angeles. *Environmental Challenges*, **9**, 100596

**Websites:**

[**https://www.afforestt.com/tinyforest**](https://www.afforestt.com/tinyforest)

Toolbox & Reports of Project “Urban Forests”: <http://urbane-waelder.de/toolbox_a.html>

General info and TF planting method by IVN: <https://www.ivn.nl/tinyforest/tiny-forest-worldwide/resources-and-downloads>

Fachverband zur Förderung der Miyawaki-Methode: <https://www.miya-forest.de/>

Urbane Waldgärten: <https://www.urbane-waldgaerten.de/>

**Videos:**

Miniwald für Klima und Natur Deshalb helfen kleine Wälder: <https://www.youtube.com/watch?v=FYAMjWB3_cE&feature=youtu.be>

The tiny urban forests bringing nature to the heart of the city - BBC World Service: <https://www.youtube.com/watch?v=y9c_Zlmqcgw&feature=youtu.be>

Tiny Forest documentary about the effects of the Miyawaki method in the Netherlands: <https://www.youtube.com/watch?v=LyHVQtDtlMk&feature=youtu.be>

**Supplementary Information 2.** Survey distributed to citizens to collect data on the perceptions around urban green spaces in the town. Presented in the original in German language (A), translated to English (B), and in the screen shots of what the survey looked like on a mobile device (C).

**A)**

1. Wo begegnet Ihnen Natur in Hallbergmoos?
2. Denken Sie bitte an Hallbergmoos. Im Folgenden sind eine Reihe von Begriffspaaren aufgeführt. Ordnen Sie bitte Ihre Eindrücke von Hallbergmoos den jeweiligen Begriffen zu.
   1. Stadt – Dorf
   2. Leise – laut
   3. Gemütlich – ungemütlich
   4. Schön – hässlich
   5. Natürlich – künstlich
   6. Grün – grau
   7. Sauber – schmutzig
   8. Persönlich – anonym
   9. Freundlich – unfreundlich
   10. Modern – altbacken
   11. Offen – verschlossen
   12. Bodenständig – abgehoben
3. Für die Zukunft von Hallbergmoos wünsche ich mir…
4. Wie sollte Ihrer Meinung nach die Grünfläche auf dem Bild gestaltet werden?
   1. Als Grünfläche erhalten
   2. Einzelne Bäume pflanzen
   3. Ein kleines Wäldchen anlegen
   4. Eine Freizeitanlage (Spielplatz, Schach, Bänke) errichten
   5. Sonstiges
5. Welche Waldform finden Sie am ansprechendsten?
   1. Nadelwald
   2. Laubwald
   3. Mischwald
6. Haben Sie einen Lieblingsort in Hallbergmoos und können Sie diesen kurz beschreiben?
7. Geschlecht:
   1. Weiblich
   2. Männlich
   3. Divers
8. Wie alt sind Sie?
   1. Unter 20
   2. 20 bis 39
   3. 40 bis 60
   4. Über 60
9. Wo verbringen Sie überwiegend Ihre Freizeit
   1. Daheim/Garten
   2. In Hallbergmoos/Sportanlagen/Grünflächen
   3. In der näheren Umgebung
   4. Weiter weg
10. Haben Sie Kinder, die in ihrem Haushalt leben?
    1. Ja
    2. Nein
11. Sind Sie Hundebesitzer?
    1. Ja
    2. Nein
12. Wenn Sie Anmerkungen zu unserer Umfrage haben oder uns etwas mitteilen wollen, können Sie dies hier tun:

**B)**

1. Where do you encounter nature in Hallbergmoos?

2. Please think of Hallbergmoos. Below are a number of pairs of terms. Please assign your impressions of Hallbergmoos to the respective terms.

a. Town - Village

b. Quiet - loud

c. Comfortable - uncomfortable

d. Beautiful - ugly

e. Natural - artificial

f. Green - gray

g. Clean - dirty

h. Personal - anonymous

i. Friendly - unfriendly

j. Modern - old-fashioned

k. Open - closed

l. Down-to-earth - aloof

3. For the future of Hallbergmoos I would like to see... (open)

4. How do you think the green area in the picture should be designed?

a. Preserve as green space

b. Plant single trees

c. Create a small grove

d. Build a recreational facility (playground, chess, benches)

e. Other

5. Which type of forest do you find most appealing?

a. Pine forest

b. Deciduous forest

c. Mixed forest

6. Do you have a favorite place in Hallbergmoos and can you describe it briefly? (open)

7. Gender:

a. Female

b. Male

c. Diverse

8. how old are you?

a. Under 20

b. 20 to 39

c. 40 to 60

d. Over 60

9. where do you spend most of your free time

a. At home/garden

b. In Hallbergmoos/sports facilities/green spaces

c. In the surrounding area

d. Further away

10. do you have children living in your household?

a. Yes

b. No

11. are you a dog owner?

a. Yes

b. No

12. if you have any comments about our survey or would like to share something with us, you can do so here:

**C)**


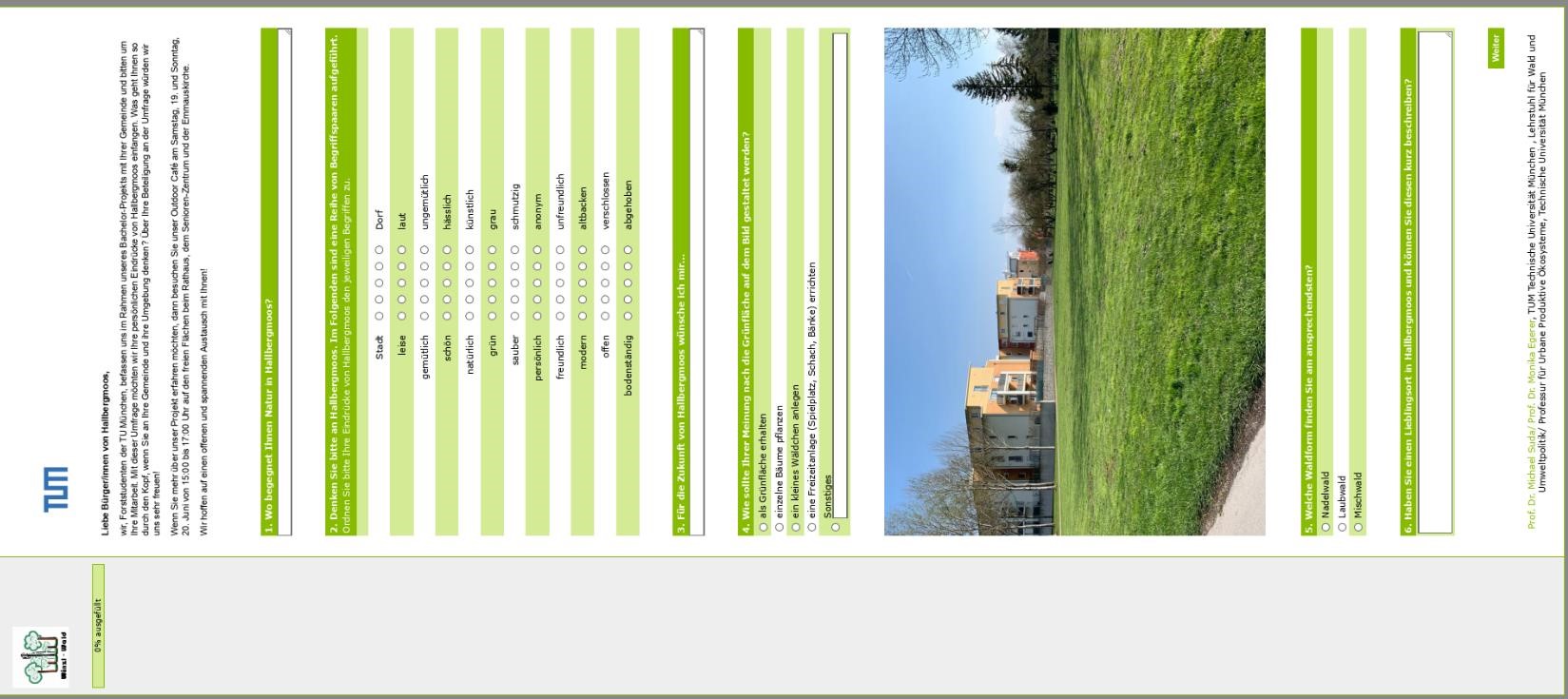

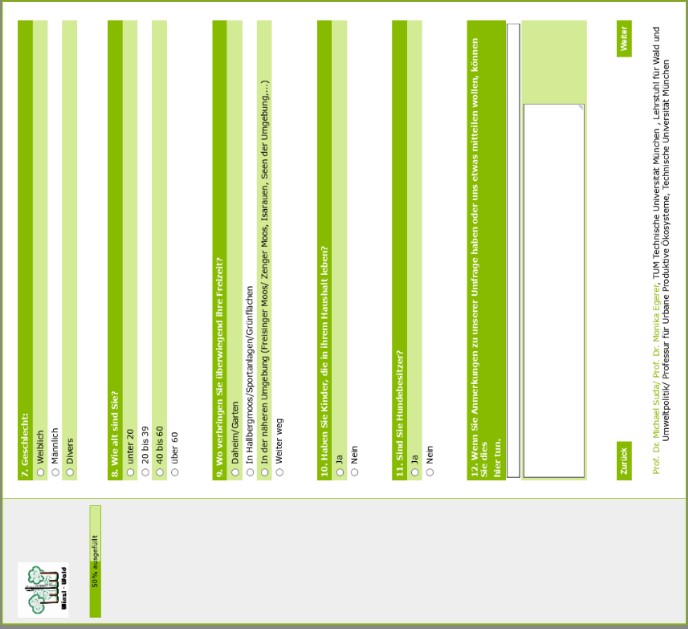


**Supplementary Information 3.** Results of the survey conducted with the municipality of Hallbergmoos.

The residents of the community were given the opportunity to talk to each other and to the students at the planned three sites on two afternoons. The event was designed as an outdoor “World Cafe". Participation was lower than anticipated due to high temperatures on both days and the European Soccer Championships. The majority of residents had a positive attitude toward the plan to create a Tiny Forest and showed interest in the implementation, expressed in numerous suggestions regarding the appearance of the forest. The residents often mentioned that they would like to see a mixed forest rich in species and structures. The forest should be visually appealing, by for example introducing flowering species or a colorful coloring of the foliage in the fall. Some fruit trees are also desired. With regard to the effects of the forest, a majority of residents reported that they would like cooling as well as shading benefits emphasized. Many residents wanted seating in the shade in the open spaces in front of the town hall, and a small path through which to walk.

In the survey questionnaire distributed across Hallbergmoos on the subject of green spaces in their home town, 130 residents responded. The following aspects stood out in particular: Goldachpark (an artificially created park along a stream) was reported as the favorite place and seems to shape the image of nature in the city. The integration of water in this park is particularly important. Overall, in the questionnaire, the idea of a Tiny Forest was accepted by residents who reported wishes and ideas, including the integration of water, the creation of meeting spaces and the preservation of existing green spaces. Overall residents reported a desire for more nature and greener meeting spaces in the town center.

**Supplementary Information 4.** Example of the pamphlet prepared by four Representatives of each working group to distribute to the municipality as a summary of the Tiny Forests project. It describes general steps to creating Tiny Forests that is easy to understand for a lay audience.
